# Draft Genome Sequence and Annotation of the Lichen-Forming Fungus Arthonia radiata

**DOI:** 10.1128/genomeA.00281-18

**Published:** 2018-04-05

**Authors:** Ellie E. Armstrong, Stefan Prost, Damien Ertz, Martin Westberg, Andreas Frisch, Mika Bendiksby

**Affiliations:** a Department of Biology, Stanford University, Stanford, California, USA; b Department of Integrative Biology, University of California, Berkeley, California, USA; c Department of Research, Botanic Garden Meise, Meise, Belgium; d Fédération Wallonie-Bruxelles, Direction Générale de l’Enseignement non Obligatoire et de la Recherche Scientifique, Brussels, Belgium; e Museum of Evolution, Uppsala University, Uppsala, Sweden; f NTNU University Museum, Norwegian University of Science and Technology, Trondheim, Norway

## Abstract

We report here the draft *de novo* genome assembly, transcriptome assembly, and annotation of the lichen-forming fungus Arthonia radiata (Pers.) Ach., the type species for Arthoniomycetes, a class of lichen-forming, lichenicolous, and saprobic Ascomycota. The genome was assembled using overlapping paired-end and mate pair libraries and sequenced on an Illumina HiSeq 2500 instrument.

## GENOME ANNOUNCEMENT

Here, we report the draft *de novo* genome assembly, transcriptome assembly, and annotation of Arthonia radiata (Pers.) Ach. (strain EZ20314). This lichen-forming fungus is the type species of Arthonia (Ach.) Ach., on which the Arthoniaceae Rchb. (Arthoniales, Arthoniomycetes), a large family of about 800 lichen-forming, lichenicolous, and saprobic Ascomycota, are based. Arthonia radiata is a common and polymorphic epiphyte on mainly smooth-barked deciduous trees throughout the Holarctic. It has also been reported in Africa and New Zealand (1). It has played a key role in recent efforts to develop a new classification of the Arthoniomycetes based on phylogenetic principles (2–4).

The genome was assembled using overlapping paired-end (PE) and mate pair (MP) libraries (with an average insert size of 5 to 8 kb) and sequenced with Illumina HiSeq 2500 v4 chemistry (2 × 125 bp). Assemblies were created using AllPaths-LG (5) and SPAdes (6). The best assembly was chosen based on assembly continuity and Benchmarking Universal Single-Copy Orthologs 2 (BUSCO2) (7) scores (using OrthoDB v9 data sets for fungi and ascomycetes, downloaded from http://busco.ezlab.org). AllPaths-LG provided the best assembly, with a contig *N*_50_ value of 1.2 Mb (46 contigs), a scaffold *N*_50_ value of 2.25 Mb (17 scaffolds), and a total sequence length of 33.5 Mb. Of all BUSCO2 genes, 99% for fungi (out of 290, none of which show duplication) and 94.8% for ascomycetes (out of 1,315, none of which show duplication) were present as complete genes in the assembly. This indicates the high quality of the presented genome.

Transcriptome assembly was performed using Trinity (8) and a combination of Hierarchical Indexing for Spliced Alignment of Transcripts v2 (HISAT2) (9) and StringTie (10). The best assembly was chosen based on assembly statistics and BUSCO2 scores. We further used different cleaning filters for read assembly with Trinity. First, we ran Trinity on the raw reads. Then, we filtered and removed only adapter sequences and low-quality bases. Last, we performed a full filtering for contaminants, adapter sequences, and low-quality bases. The combination of AdapterRemoval (11) and Trinity resulted in the best transcriptome assembly, with 27,220 contigs and an *N*_50_ value of 6.8 kb. BUSCO2 scores indicated the presence of 94% complete and 5.5% fragmented transcripts (2 out of 290 are missing) for fungal BUSCO2 genes and 92.5% complete and 5.5% fragmented transcripts for ascomycete BUSCO2 genes (26 out of 1,315 are missing).

Next, we annotated repeats in the best genome assembly using a combination of RepeatMasker (homology-based annotation using the Repbase database) and RepeatModeler (*de novo* repeat annotation) (see http://repeatmasker.org for both programs). We found that 16.65% of the *A. radiata* genome constitutes repeat sequences, most of which were LTR elements (14.94% of the whole genome).

Gene annotation was performed using Maker3 (12), with simple repeats only soft masked in the repeat-masking step. We annotated 6,931 genes.

### Accession number(s).

This whole-genome shotgun project has been deposited at GenBank (assembly number GCA_002989075), and all the data are available at NCBI (BioProject number PRJNA432823, BioSample number SAMN08462631) under the accession number PSQN00000000 (locus numbers PSQN01000001 to PSQN01000017).
